# *Pasteurella* lipoprotein E interacts with host ABCF2 to facilitate adhesion and cell apoptosis

**DOI:** 10.1002/mlf2.70056

**Published:** 2026-07-19

**Authors:** Yuyao Shang, Fei Wang, Menghan Chen, Zihao Wang, Hanyuan Liu, Lin Hua, Huanchun Chen, Bin Wu, Zhong Peng

**Affiliations:** ^1^ National Key Laboratory of Agricultural Microbiology, College of Veterinary Medicine Huazhong Agricultural University Wuhan China; ^2^ Hubei Hongshan Laboratory Wuhan China; ^3^ Frontiers Science Center for Animal Breeding and Sustainable Production The Cooperative Innovation Center for Sustainable Pig Production Wuhan China

**Keywords:** ABCF2, p53‐dependent apoptosis, *Pasteurella multocida*, PlpE, TurboID‐based proximity labeling

## Abstract

*Pasteurella multocida* can cause serious diseases in many animal species and humans, yet the mechanisms of its pathogenesis remain to be elucidated. In this study, we found that the lipoprotein E (PlpE) of *P. multocida* contributed to bacterial adhesion to host cells. Through turboID‐based proximity labeling screening, we identified ATP‐binding cassette sub‐family F member 2 (ABCF2) as a host‐interaction protein for PlpE, and Asp‐123 in PlpE was identified as the crucial amino acid residue for the interaction. Knocking out ABCF2 significantly reduced the adhesion of *P. multocida* to host cells. Additionally, we demonstrated that *P. multocida* infection upregulated the expression of host ABCF2 by activating the NF‐κB signaling. Furthermore, we showed that ABCF2 was involved in the *P. multocida*‐induced p53‐dependent apoptotic signaling pathway. To the best of our knowledge, this is the first identification of ABCF2 as a host factor contributing to the adhesion of *P. multocida* and only the second report of ABCF2's involvement in bacterial pathogenesis.

## Impact statement

*Pasteurella multocida* can cause fatal infections in both animals and humans. This study uncovers a previously unrecognized pathogenic mechanism of *P. multocida* infection, demonstrating the role of PlpE in facilitating the bacterium's adhesion to host cells through interaction with the host protein ABCF2. *P. multocida* infection boosts ABCF2 to increase its stickiness and accelerate cell death. This study could be valuable for comprehending the interactions between bacteria and hosts during infections.

## INTRODUCTION

Adhesion of pathogens to host cells or tissues is a universal prerequisite for bacteria to efficiently deploy their repertoire of virulence factors and cause infections^1^. Consequently, bacteria have evolved various virulence factors to assist in binding to host cells during infection, including capsules, lipopolysaccharides (LPS), fimbriae, pili, and outer membrane proteins (OMPs)^2^. On the surface of host cells, there exist numerous known or unknown receptors that can be recognized by bacterial adhesive proteins to establish interactions^3^. Identifying important bacterial adhesive proteins and their corresponding host receptors is not only crucial for understanding bacterial pathogenesis but also aids in the development of anti‐adhesion‐based therapies for bacterial diseases^4^.

*Pasteurella multocida*, a Gram‐negative zoonotic bacterium that can cause fatal diseases in humans and a wide range of animals, poses a significant threat to public health, leading to high morbidity, mortality, and economic losses in agriculture^5^. Clinical strains of *P. multocida* are classified into five capsular serogroups (A, B, D, E, and F)^6^. Among these serogroups, types A and/or D are predominantly prevalent in various host species^7^, and frequently lead to respiratory symptoms^5^. Indeed, *P. multocida* strains have been found to be able to disrupt the mammalian respiratory epithelial barrier^8^. However, the interactions between *P. multocida* and hosts remain to be explored.

Bacterial lipoproteins represent a subset of membrane proteins localized to either leaflet of the lipid bilayer^9^. These proteins play diverse critical roles in bacterial physiology and virulence, including nutrient uptake, cell wall metabolism, cell division, transmembrane signal transduction, antibiotic resistance, and adhesion to host tissues during infection^10^. While several lipoproteins have been identified in *P. multocida* through bioinformatic or chemical strategies^11^, their functions contributing to the fitness and pathogenesis of *P. multocida* are still unclear. Recently, a surface lipoprotein PmSLP of *P. multocida* was found to be able to bind host complement factor I (FI) to promote immune evasion^12^. In addition to PmSLP, many clinical strains of *P. multocida* encode a 39 kDa lipoprotein named *Pasteurella* lipoprotein E (PlpE)^11^. This highly conserved lipoprotein (90%–100% amino acid similarities between different strains) has been found to show good immunogenicity and could provide good protections against *P. multocida* infection^13^, ^14^, ^15^, suggesting that it might play an important role in the pathogenesis of *P. multocida*. In this study, we showed that PlpE contributed to the adhesion of *P. multocida*. By employing TurboID‐based proximity labeling, which has emerged as a novel approach for studying the spatial and interaction characteristics of proteins in living cells^16^, we identified the ATP‐binding cassette sub‐family F member 2 (ABCF2) as an adhesion receptor for *P. multocida* on mammalian respiratory epithelial cells. In particular, we found that ABCF2 is involved in the *P. multocida*‐induced p53‐dependent apoptotic signaling pathway.

## RESULTS

### PlpE contributes to *P. multocida* adhering to host cells

**Figure 1:**
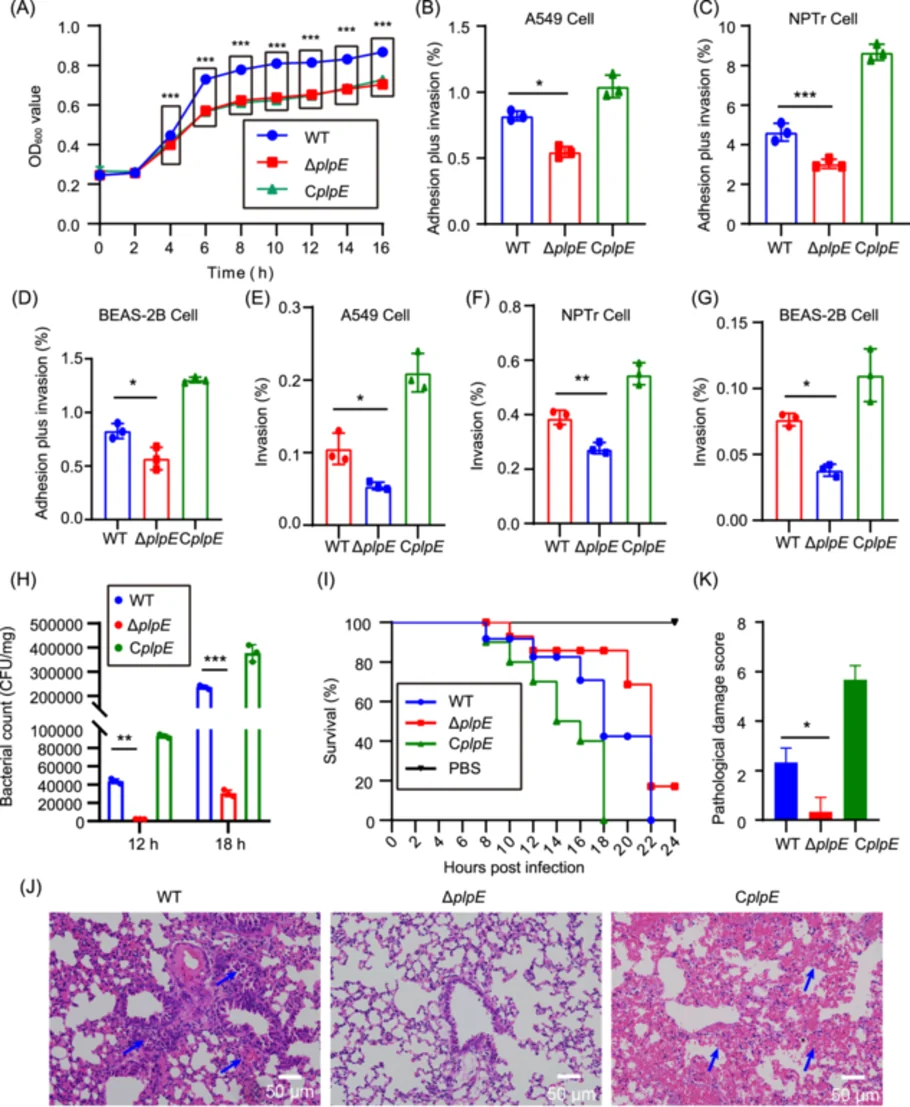
Assessments of the impact of PlpE on the virulence of *Pasteurella multocida*. (A) Growth curves of *P. multocida* wild‐type strain (WT), *plpE*‐deleted strain (Δ*plpE*), and *plpE* complementary strains (C*plpE*) in tryptic soy broth (TSB) containing 5% newborn calf serum. (B–D) Column charts depicting the percentage of *P. multocida* strains adhering to and invading A549 cell (B), NPTr cell (C), and BEAS‐2B cell (D). (E–G) The percentage of *P. multocida* strains invading A549 cell (E), NPTr cell (F), and BEAS‐2B cell (G). (H) Colony‐forming unit (CFU) counts of *P. multocida* WT, Δ*plpE*, or C*plpE* strains recovered from murine lungs at 12 and 18 h post infection (hpi). (I) Survival curves of mice challenged with PBS, *P. multocida* WT, Δ*plpE*, or C*plpE* strains. (J) Histopathological examinations were performed on lung tissues collected from mice at 12 hpi. Lung damage features, including thickened/collapsed alveolar walls, erythrocyte diapedesis, and inflammatory cell infiltration, are marked with blue arrows. Scale bar, 50 μm. (K) Pathological damage scores of mouse lung tissues at 12 hpi. Data are presented as the mean ± standard deviation (SD). Differences between two groups were analyzed by unpaired *t*‐test. *p < 0.05; **p < 0.01; and ***p < 0.001.

To explore the functional role of PlpE, we deleted *plpE* from the *P. multocida* strain HuN001 (GenBank accession No. CP073238) (Figure S1A,B) and constructed a gene‐complementary strain (C*plpE*) following a protocol described previously^17^. Measurement of growing curves showed that deletion of *plpE* significantly decreased the growth of *P. multocida* compared to the wild‐type strain (Figure 1A). Cell adhesion and invasion assays revealed that the *plpE*‐deleted strain (Δ*plpE*) exhibited significantly reduced cell adhesion and/or invasion capabilities compared to the wild‐type strains in different cell models (Figure 1B–G). *In vivo* tests in mouse models showed that Δ*plpE* exhibited a significantly decreased capacity for adhering to and invading murine lungs compared to the wild‐type strain (Figure 1H). Although Δ*plpE* also induced murine death after challenge, its capacity to induce pathological damage in murine lungs was significantly attenuated compared to the wild‐type strain (Figure 1I–K). These findings revealed that PlpE contributed to the adhesion and virulence of *P. multocida*.

### ABCF2 is identified as a host factor to interact with PlpE to mediate *P. multocida* adhesion

**Figure 2:**
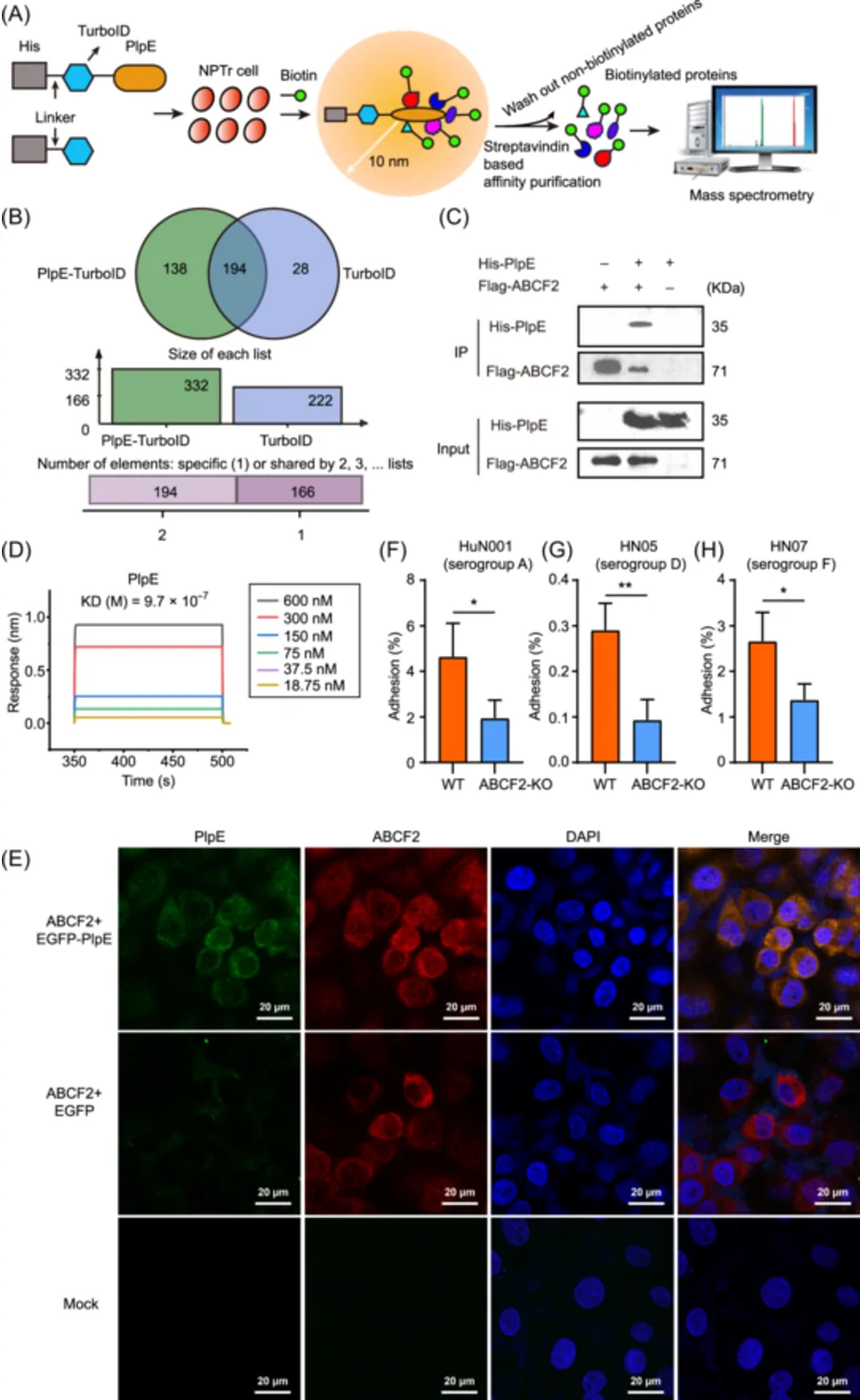
TurboID screening identifies ABCF2 as a host protein interacting with *P. multocida* PlpE. (A) Schematic workflow of TurboID screening. (B) Venn diagram illustrating overlapping and unique proteins captured by recombinant PlpE‐TurboID versus TurboID controls. A total of 332 proteins were captured in the PlpE‐TurboID group and 222 proteins in the control group, with 194 common proteins identified in both datasets. There are 166 unique proteins identified to be specific for the PlpE‐TurboID group and the TurboID group. (C) Co‐immunoprecipitation (Co‐IP) validation of ABCF2–PlpE interactions. (D) Bio‐layer interferometry (BLI) assays confirming ABCF2‐PlpE binding kinetics. The equilibrium dissociation constant (KD) was determined to be 9.7 × 10^−7^ M. (E) Confocal immunofluorescence showing the colocalization of ABCF2 (red) and PlpE (green). Scale bar, 20 μm. (F–H) Adherence percentages of *P. multocida* serogroup A (F), serogroup D (G), and serogroup F (H) to ABCF2 knockout (ABCF2‐KO) or WT cells. Data are presented as the mean ± standard deviation (SD). Differences between two groups were analyzed by unpaired *t*‐test. *p < 0.05; **p < 0.01.

Subsequently, we utilized TurboID‐based proximity labeling to screen for host receptors for *P. multocida* PlpE (Figure 2A), following a previously established protocol^18^. Mass spectrometric analysis identified numerous host effector proteins potentially interacting with *P. multocida* PlpE in swine trachea epithelial cells NPTr (Figure 2B), but ABCF2 was consistently present in the candidate lists with a high score (iBAQ value: 475810). Therefore, we selected this host protein for further investigation. Immunofluorescence assay (IFA) showed that ABCF2 was mainly expressed in the cytoplasm of A549 cells (Figure S1C). Further analyses using co‐immunoprecipitation (Co‐IP) and bio‐layer interferometry (BLI) revealed clear interactions between ABCF2 and *P. multocida* PlpE (Figure 2C,D). Additionally, colocalizations were observed between ABCF2 and PlpE (Figure 2E).

To evaluate the impact of ABCF2 on *P. multocida* adhesion, we employed an *ABCF2*‐knockout (ABCF2‐KO) A549 cell line to assess bacterial adhesion using *P. multocida* strains of different serogroups (types A, D, and F). Before that, we evaluated the influence of *ABCF2*‐KO on the growth of the A549, and the results showed that ABCF2‐KO decreased the growth of the cells from 3 days post‐inoculation (Figure S1D). Therefore, we adjusted the cell quantity and conducted the bacterial adhesive assays. The results revealed that ABCF2‐KO significantly decreased the quantity of different serogroups of *P. multocida* strains adhering to host cells (Figure 2F–H). These findings emphasize the essential role of ABCF2 in mediating *P. multocida* adhesion to host cells.

### Asp at site 123 in PlpE is the key amino acid for interacting with ABCF2

**Figure 3:**
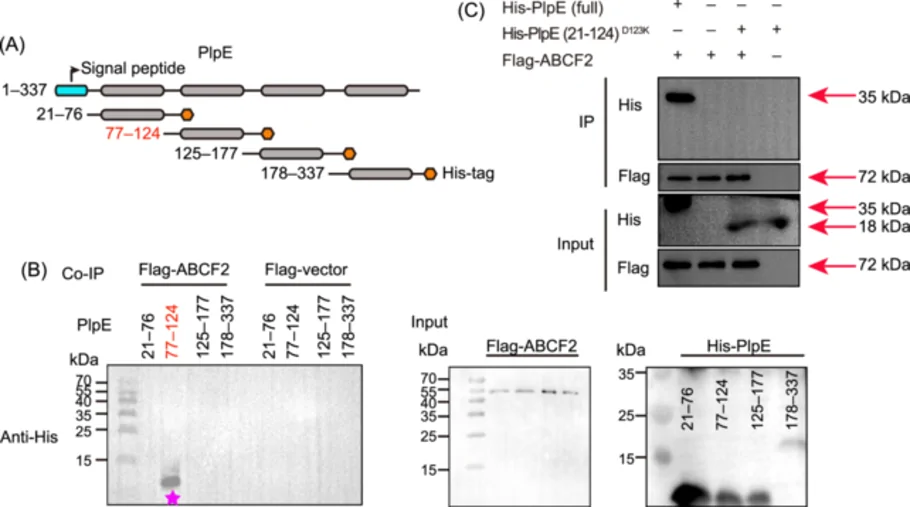
Preliminary verification of amino acid residues in *P. multocida* PlpE crucial for interacting with ABCF2. (A) Schematic strategy for designing truncated PlpE proteins containing the identified amino acid residues, based on molecular docking analysis. (B) Validation of interactions between ABCF2 and PlpE‐derived truncated proteins via Co‐IP assays. (C) Validation of interactions between ABCF2 and PlpE‐derived proteins carrying the D123K amino acid mutation via Co‐IP assays.

To further explore the interactions between ABCF2 and *P. multocida* adhesion proteins, we conducted molecular docking analysis and utilized AlphaFold3^19^ to predict the essential amino acids in PlpE involved in its interaction with ABCF2. This computational approach unveiled a set of key amino acids deemed crucial for the interaction between PlpE and ABCF2. These residues encompassed Asp123, Ser186, Glu321, Thr292, Asn296, and Thr298 (Figure S1E). Subsequently, we generated and expressed a series of truncated proteins containing the identified amino acid residues, and evaluated their interactions with ABCF2 through Co‐IP assays (Figure 3A,B). The outcomes highlighted that Asp‐123 in PlpE might be a key site for interacting with ABCF2. Further analysis through Co‐IP confirmed the critical role of Asp‐123 in interacting with ABCF2 (Figure 3C).

### Knockout of *plpE* decreases the expression of ABCF2 promoted by *P. multocida* infection

**Figure 4:**
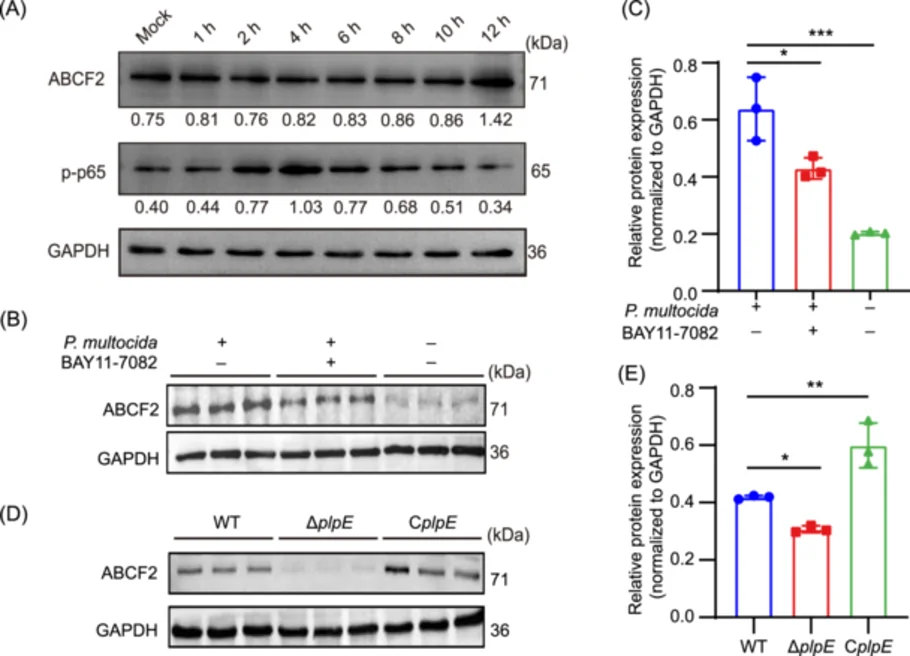
*P. multocida* infection upregulates ABCF2 expression via the NF‐κB signaling. (A) Western blot analysis of ABCF2 and p‐p65 expression in NPTr cells at 0 (mock), 1‐, 2‐, 4‐, 6‐, 8‐, 10‐, and 12‐hpi with *P. multocida*. (B) Western blot analysis of ABCF2 expression in NPTr cells treated with (+) or without (–) NF‐κB inhibitor BAY11‐7082, followed by P. multocida infection for 12 h. (C) Quantification of Western blot bands in (B) using ImageJ software. (D) Western blot analysis of ABCF2 expression in NPTr cells infected with *P. multocida* WT, Δ*plpE*, or C*plpE* for 12 h. (E) Quantification of Western blot bands in (D) using ImageJ software. Data are presented as mean ± SD. Statistical comparisons were performed using one‐way ANOVA followed by Tukey's post hoc test. *p < 0.05; **p < 0.01; and ***p < 0.001.

Given the reported regulation of ABCF2 by the nuclear factor‐kappa B (NF‐κB) signaling pathway in LPS‐induced ulcerative colitis models^20^, we explored the impact of NF‐κB on ABCF2 regulation post *P. multocida* infection. The findings revealed a notable increase in p65 phosphorylation (p‐p65) between 2 hours post infection (hpi) and 10 hpi with *P. multocida* (Figure 4A). Treatment with the NF‐κB inhibitor (BAY11‐7082) markedly reduced the expression of ABCF2 induced by *P. multocida* infection (Figure 4B,C), indicating the involvement of NF‐κB in regulating ABCF2 expression post *P. multocida* infection. However, knockout of *plpE* led to a significant decrease in ABCF2 expression (Figure 4D,E). These findings indicate that PlpE participates in ABCF2 expression accelerated by *P. multocida* infection by elevating the NF‐κB.

### ABCF2 is involved in the *P. multocida‐*induced p53‐dependent apoptotic signaling pathway

**Figure 5:**
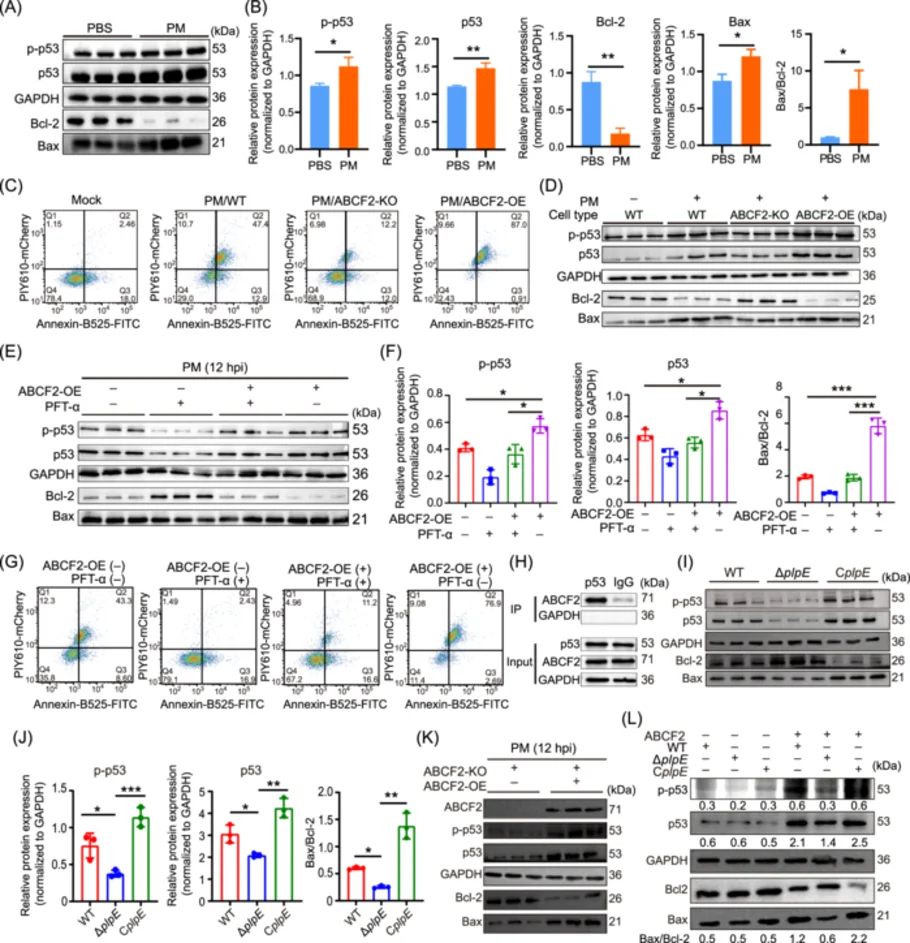
Involvement of ABCF2 in *P. multocida‐*induced p53‐dependent apoptosis. (A) Western blot analysis of p53, p‐p53, Bcl‐2, and Bax protein levels in NPTr cells at 12 hpi with *P. multocida* (PM). (B) Quantification of Western blot bands in (A) using ImageJ software. (C) Flow cytometry analysis of apoptotic cells in A549 wild type cells, ABCF2‐KO cells, and ABCF2‐overexpressing (ABCF2‐OE) cells at 8 hpi with PM. (D) Western blot analysis of p53, p‐p53, Bcl‐2, and Bax levels in A549 wild‐type cells, ABCF2‐KO cells, and ABCF2‐OE cells at 12 hpi with PM. (E) Western blot analysis of p53, p‐p53, Bcl‐2, and Bax protein levels in ABCF2‐overexpressing (ABCF2‐OE) A549 cells treated with (+) or without (−) p53 inhibitor pifithrin‐α (PFT‐α), following 12 hpi with PM. (F) Quantification of the Western blot bands in (E) using ImageJ software. (G) Flow cytometry analysis of apoptotic cells in A549 ABCF2‐OE cells treated with (+) or without (−) PFT‐α, at 8 hpi with PM. (H) Co‐IP assay showing the endogenous interaction between ABCF2 and p53 in A549 cells. (I) Western blot analysis of p‐p53, p53, Bcl‐2, and Bax protein levels in A549 cells infected with different PM strains (WT, Δ*plpE*, and *CplpE*) at 12 hpi. (J) Quantification of the Western blot bands in (I) using ImageJ software. (K) Western blot analysis of p‐p53, p53, Bcl‐2, and Bax protein levels in ABCF2‐KO and ABCF2‐OE A549 cells at 12 hpi with PM. (L) Western blot analysis of p‐p53, p53, Bcl‐2, and Bax protein levels in ABCF2‐KO and ABCF2‐OE A549 cells infected with different PM strains (WT, Δ*plpE*, and *CplpE*) at 12 hpi. In all bar graphs, data are presented as the mean ± SD. Statistical comparisons were performed by unpaired *t*‐test (B) or using two‐way ANOVA test (F,J). *p < 0.05; **p < 0.01; and ***p < 0.001.

Given the well‐documented association between ABCF2 and apoptosis^21^, ^22^, we investigated the impact of *P. multocida* infection on cell apoptosis. Analyses of cell apoptosis marker (Bcl‐2 and Bax) expression and/or cleavage, along with annexin V staining, revealed that *P. multocida* infection promoted apoptotic cell formation, upregulated the proapoptotic protein Bax, and downregulated the expression of the antiapoptotic protein Bcl‐2 (Figure 5A–C). These findings indicated that *P. multocida* infection indeed induced cell apoptosis. Subsequently, we assessed cell apoptosis in ABCF2‐overexpressed and knockout cells post *P. multocida* infection. The results showed that ABCF2 overexpression elevated the level of cell apoptosis, while ABCF2 knockout significantly reduced the level of cell apoptosis induced by *P. multocida* infection (Figures 5C–E and S2), suggesting that ABCF2 contributes to *P. multocida*‐induced cell apoptosis.

**Figure 6:**
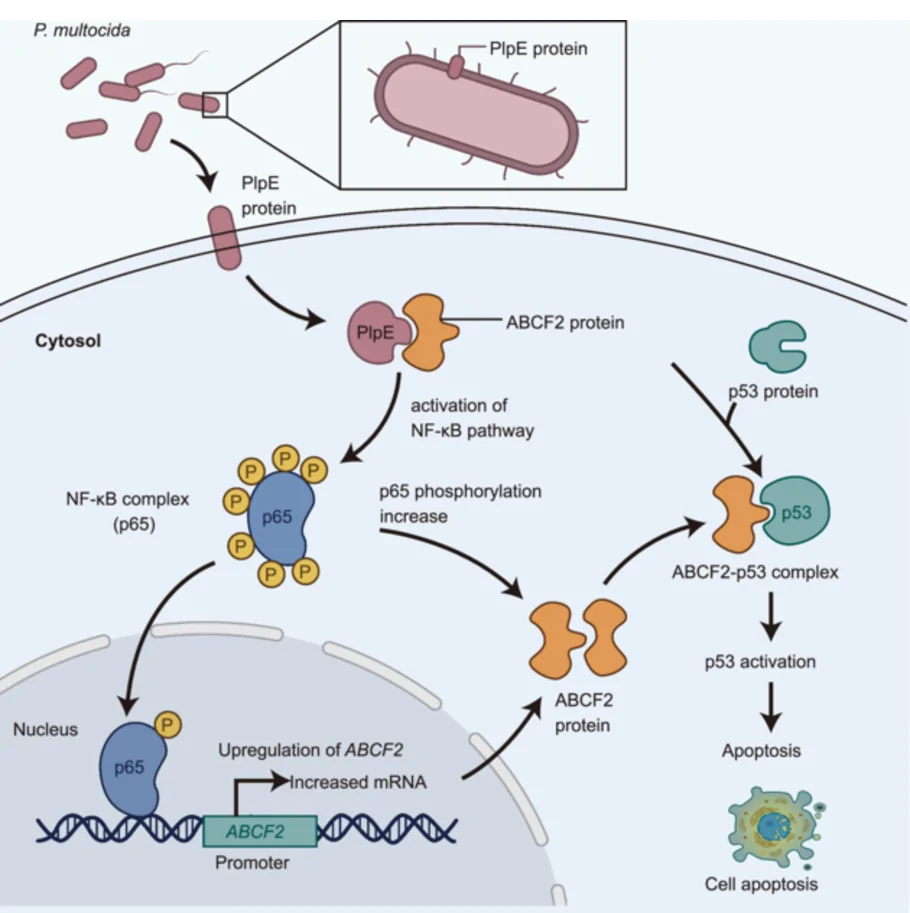
Schematic model depicting interactions between *P. multocida* PlpE and host ABCF2. Following infection, *P. multocida* upregulates ABCF2 in host cells via activation of the NF‐κB signaling pathway. This upregulated ABCF2 facilitates bacterial adhesion to and invasion to host cells by interacting with PlpE. Elevated ABCF2 levels further promote p53‐dependent apoptosis during *P. multocida* infection.

Upon reviewing mass spectrometry data published in a recent article^23^, we observed an association between ABCF2 and p53. Given the well‐established role of p53‐dependent apoptosis, particularly in cancer^24^, ^25^, we further investigated whether p53 was involved in ABCF2‐induced cell apoptosis following *P. multocida* infection. Our analysis revealed that *P. multocida* infection significantly increased the expression and phosphorylation of p53 (p‐53) (Figures 5A,B,D,E and S2). Furthermore, overexpression of ABCF2 notably enhanced the expression and phosphorylation of p53 induced by *P. multocida*, while ABCF2 knockout significantly decreased these levels (Figures 5D and S2). Consistently, changes in the levels of cell apoptosis markers (Bcl‐2 and Bax) and apoptotic cells aligned with these observations (Figures 5D–F and S2). Additionally, inhibition of p53 in wild‐type cells or ABCF2‐overexpressed cells also reduced the incidence of cell apoptosis (Figure 5E–G), indicating that p53 and its phosphorylation played a role in ABCF2‐induced cell apoptosis post *P. multocida* infection. Further analysis revealed an interaction between ABCF2 and p53 (Figure 5H). It was also found that deletion of *plpE* decreased p‐p53 and ABCF2‐associated cell apoptosis induced by *P. multocida* infection (Figure 6I–L).

## DISCUSSION

As a long‐recognized zoonotic pathogen (confirmed in 1930), *P. multocida* has been responsible for an increasing number of human infections in recent years^26^, ^27^. However, our understanding of the pathogenesis of *P. multocida* remains limited. Numerous review articles and genome‐based studies have highlighted various virulence factors that potentially contribute to the pathogenesis of *P. multocida*, with bacterial adhesion‐associated proteins being a significant focus^5^, ^7^, ^11^, ^28^, ^29^. In this study, we found that PlpE plays a role in the adhesion of *P. multocida* to hosts. PlpE was initially identified in *Mannheimia haemolytica* (formerly *Pasteurella haemolytica*), a bacterial species closely related to *P. multocida* ^30^. The homolog of this protein was subsequently discovered in the outer membrane of *P. multocida*, categorizing it as a type of *P. multocida* outer membrane protein^11^. Despite the detailed function of this protein not being fully elucidated, several studies have indicated that recombinantly expressed PlpE exhibits significant immunogenic effects^15^, ^31^. Our study findings suggest that PlpE may also function as a bacterial adhesion‐related protein in *P. multocida*. In addition, our results also showed that deletion of *plpE* led to a significant decrease in the growth of *P. multocida*. This might be partly because PlpE is a part of the outer membrane of *P. multocida* ^11^, and its loss may decrease the bacterial membrane integrity. The loss of bacterial membrane integrity has been shown to lead to slow growth and even bacterial death^32^, ^33^. A slower growth of Δ*plpE* may also result in a lower bacterial load in murine lungs compared to the wild‐type strains.

In this study, we identified ABCF2 as a host protein that plays roles in the adherence and pathogenesis of *P. multocida*. ABCF2 belongs to the superfamily of ATP‐binding cassette (ABC) transporters, and its functions have been extensively investigated in the field of oncology^21^, ^34^, ^35^. Before this report, only one study had reported the involvement of ABCF2 in bacterial pathogenesis^22^. In that study, ABCF2 was identified as a target of EspF linked to apoptosis during infections with Enteropathogenic *E. coli* (EPEC)^22^. However, unlike the study that observed an antiapoptotic and protective role against various apoptotic stimuli for ABCF2 during EPEC infection^22^, we demonstrated that ABCF2 had a proapoptotic role during *P. multocida* infection. Furthermore, we found an increased levels of ABCF2 in cells infected with *P. multocida* wild‐type cells and C*plpE* compared to those in cells infected with Δ*plpE*. This differs from the EPEC study, which found the *espF* mutant EPEC strain induced increased level of ABCF2 in cells compared to the wild‐type strains and complemented strains^22^, might be partly owing to the utilization of different types of cells and/or the different interactions between ABCF2 and different bacterial effector proteins. In the EPEC study, two types of cells (HeLa and Caco‐2) were used, and the investigated bacterial protein was EspF, which is not an outer membrane protein but a secreted protein of unknown function injected from EPEC into host cells via a type III secretion system^22^, ^36^. It was suggested that ABCF2 may have diverse associations with cell apoptosis during bacterial infections.

Our analysis also revealed that *P. multocida* infection upregulates the expression level of ABCF2 by activating the NF‐κB signaling. The regulation of ABCF2 by the NF‐κB signaling has been reported in LPS‐induced ulcerative colitis^20^. Being a pathogen commonly associated with bacterial pneumonia and other inflammatory disorders, *P. multocida* infection is consistently accompanied by the activation of NF‐κB signaling, a well‐known proinflammatory pathway^37^, ^38^. Recently, *P. multocida* infection has been found to induce cell apoptosis^39^, ^40^. In this study, we demonstrated that ABCF2 is associated with p53‐dependent apoptosis induced by *P. multocida* infection. Notably, p53‐dependent apoptosis has also been observed in infections caused by various other bacterial species, including *Haemophilus parasuis*, a species closely related to *P. multocida* ^41^. However, the connection between ABCF2 and p53‐dependent apoptosis in bacterial infections has not been reported previously. In numerous noninfectious disease models, p53 has been shown to be activated by inflammatory factors and/or reactive oxygen species (ROS)^42^, ^43^, leading to its upregulation at sites of inflammation^44^. Given that *P. multocida* infection typically triggers inflammation in multiple organs, such as the lungs and brain, and can also induce ROS production in the lungs^8^, ^37^, p53‐dependent apoptosis may play a significant role in *P. multocida* pathogenesis.

In summary, we have elucidated the role of PlpE as an adhesive protein for *P. multocida* and identified ABCF2 as a host protein that interacts with PlpE. Upon infection, *P. multocida* upregulates ABCF2 through activation of the NF‐κB signaling pathways. This upregulation of ABCF2 facilitates the adhesion and invasion of host cells by *P. multocida*. Moreover, elevated levels of ABCF2 are linked to p53‐dependent apoptosis following *P. multocida* infection (Figure 6). The findings presented in this study not only deepen our comprehension of the pathogenesis of the zoonotic pathogen *P. multocida* but also emphasize the involvement of ABCF2 and ABCF2‐mediated p53‐dependent apoptosis in bacterial infections.

## MATERIALS AND METHODS

### Bacterial strains, cell lines, and culture conditions

*P. multocida* strain HuN001 (GenBank accession No. CP073238, serogroup A) from a pneumonia patient's sputum^45^ was used alongside strains HN05 (GenBank accession No. PPVF01000051, serogroup D), and HN07 (GenBank accession No. CP007040, serogroup F)^46^. *P. multocida* strains were cultivated on tryptic soy agar (TSA) or in tryptic soy broth (TSB) (BD) with 5% newborn calf serum (Tianhang). NPTr cells, A549 cells, and human tracheal epithelial cells BEAS‐2 B were maintained as previously described^8^, ^37^.

### Construction of bacterial gene deletion and complementary strains

The *plpE* gene was knocked out in *P. multocida* strain HuN001 following a known procedure^17^. Upstream and downstream *plpE* fragments were PCR‐amplified using specific primers (Table S1), cloned into plasmid pSHK5(TS)‐NgAgo to form pSHK‐Δ*plpE*, and transformed into HuN001 cells via electroporation (2500 V, 25 μF, 600 Ω). Cultures were grown in TSB with 10% calf serum at 28°C for 3 h, then plated on TSA with kanamycin (50 μg/ml) and serum (5%) for selection. PCR confirmed initial gene replacement with primers *plpE*‐oL/*plpE*‐oR (Table S1), followed by overnight culture on TSA at 42°C. Deletion was confirmed via PCR using primers *plpE*‐iL/iR (Table S1) and Sanger sequencing. For complementation, the full *plpE* sequence was PCR‐amplified using primers C‐*plpE*‐F/C‐*plpE*‐R (Table S1), inserted into pPBA1101 (gifted by Prof. John Boyce at Monash University), and transformed into HuN001‐Δ*plpE* cells through electroporation.

### TurboID screening

To identify host proteins interacting with PlpE from *P. multocida*, its nucleotide sequence was PCR‐amplified with specific primers (Table S1). The turboID sequence was also amplified from pET28a‐ompP2‐turboID (gifted by Prof. Wentao Li at Huazhong Agricultural University) and linked to *plpE* fragment via overlap PCR, then cloned into pET‐28a (+) to create a recombinant plasmid. *E. coli* BL21 cells were transformed with these plasmids and induced to express the proteins using IPTG (0.8 mM; Sigma). The recombinant proteins were purified using HisTrap HP columns (GE HealthCare). Next, purified proteins (His‐TurboID‐PlpE, and/or His‐TurboID; 3 μg) were incubated with NPTr cells on ice for 1 h, followed by biotin (50 μM; Sigma) addition and incubation at 37°C for 15 min. After cell lysis using a cell lysis buffer for Western blot and IP containing a protease inhibitor cocktail (Beyotime), the lysate was centrifuged, and supernatants were incubated with streptavidin magnetic beads (NEB) overnight at 4°C. Biotinylated proteins were eluted by boiling the beads in 100 μl of SDT buffer (4% SDS, 100 mM Tris‐HCl, 1 mM DTT, pH 7.6) and analyzed by mass spectrometry to identify host cell‐interacting proteins. The mass spectrometry proteomics data have been deposited in the ProteomeXchange Consortium via the iProX partner repository^47^ under the dataset identifier PXD065317.

### Immunofluorescence assay (IFA)

To investigate the localization of ABCF2, monolayers of A549 cells in wells of a 6‐well plate (Corning) were washed using PBS three times and were fixed using 4% paraformaldehyde (Sigma) for 30 min. After washing again using PBS, the cells were blocked in 5% skimmed milk for 2 h at room temperature. The cells were co‐incubated with ABCF2 polyclonal antibody (Cat No. 10226‐1‐AP, 1:500; Proteintech) for overnight at 4°C. After washing, the cells were incubated with a mixture of goat anti‐mouse IgG H&L (Alexa Fluor® 488) (Cat No. ab150113, 1:1000; Abcam) and Cy3–conjugated goat anti‐rabbit IgG (H + L) (Cat No. SA00009‐2, 1:200; Proteintech) for 1 h at 37°C and were stained using DAPI (Beyotime) for 5 min. Finally, the localization of ABCF2 in A549 cells was observed using a Nikon AX/AX R Confocal Microscope System (Nikon).

### Cell proliferation assay

To investigate the impact of ABCF2‐KO on cell proliferation, monolayers of ABCF2‐KO cells and A549 cells harboring the nontarget control sgRNA (NTC cells) were inoculated in wells (100 μl, approximately 5000~6000 cells per well) of a 96‐well plate (Corning) and were cultured under 5% CO_2_ at 37°C for 0, 1, 2, 3, 4, and 5 days. Each day, cells in each well were incubated with 10 μl of Enhanced Cell Counting Kit‐8 reagent (Cat No. C0041; Beyotime) for 2 h, and the growth condition of cells was indicated by measuring the absorbance values at 450 nm (OD_450_) using a microplate reader (Bio‐Rad), as described previously^48^.

### Cell adhesion and invasion assays

To assess the impact of PlpE on bacterial adherence and invasion, different cells (A549, NPTr, and BEAS‐2B) cultured in wells (2.5 × 10^6^ cells per well) of a 96‐well plate (Corning) were infected with *P. multocida* wild‐type, Δ*plpE*, and C*plpE* strains at an optimal multiplicity of infection (MOI) of 200, respectively. After incubation at 37°C for 1.5 h, the cells were washed using PBS and then lysed in 1 ml of sterile distilled water. To quantify the adherent bacteria, the cells were treated with gentamicin (100 μg/ml) and incubated at 37°C for 1 h before lysis. Serial 10‐fold dilutions of the lysed cells were plated on TSA containing 5% newborn calf serum for bacterial enumeration. The number of adherent bacterial strains was calculated as the ratio of the bacterial counts from the lysed cells without antibiotic treatment to those with antibiotic treatment. These experiments were independently repeated three times.

The contribution of ABCF2 to *Pasteurella* adhesion was assessed using an ABCF2‐KO A549 cell line stored at our laboratory, and the wild‐type A549 cells, which were inoculated with *P. multocida* strains HuN001, HN05, and HN07, as described above.

### Molecular docking

Protein‐protein interactions were explored via molecular docking to pinpoint crucial amino acids. The structures of PlpE and ABCF2 were modeled using AlphaFold3 and visualized with PyMOL 3.03. During docking, ABCF2 acted as the receptor, while PlpE was the ligand. Schrödinger software facilitated docking following a standard protocol^49^. Truncated proteins were crafted based on essential amino acids from docking results, and mutations were made with the Mut Express II Fast Mutagenesis Kit V2 (Vazyme). These variants were expressed in *E. coli* BL21, and purification was followed using GE Healthcare HisTrap HP columns (GE HealthCare) to delve deeper into their interactions and functions.

### Co‐IP

To investigate the interaction between ABCF2 and PlpE and its mutant proteins, pCAGGS‐N‐flag‐ABCF2‐P and pCAGGS‐N‐flag were transfected into HEK293T cells using Lipofectamine 2000. After a 24‐h incubation at 37°C, cells were lysed with cell lysis buffer (Beyotime) for Western blot and Co‐IP, and proteins (2 mg) were incubated with His‐tagged proteins (PlpE and/or mutant proteins, 2.5 μg) for 2 h at 4°C (step 1). Concurrently, a commercially purchased DYKDDDDK‐tagged mouse monoclonal antibody (2.5 μg, Proteintech) was incubated with rProtein A/G Magarose Beads (2.5 μl; Smart‐Lifesciences) for 4 h at room temperature to obtain DYKDDDDK‐tagged beads. Subsequently, the protein complex from step 1 was incubated with the DYKDDDDK‐tagged beads overnight at 4°C. The beads were then incubated in 3× Flag Peptide (Beyotime) for 2 h at 4°C to elute the proteins, which were later separated by SDS‐PAGE. Proteins were transferred onto a PVDF membrane (Bio‐Rad) and blocked in PBST containing 5% BSA for 3 h at room temperature. The blots were probed with the DYKDDDDK‐tagged mouse monoclonal antibody (1:5000; Proteintech), His‐tag polyclonal antibody (1:5000; Proteintech), and/or GAPDH polyclonal antibody (1:5000; Proteintech) overnight at 4°C. After washing using PBST, the blots were incubated with HRP‐conjugated goat anti‐mouse IgG(H + L) (1:5000; Proteintech) for 1 h, followed by being visualized using BeyoECL Star (catalog No. SA00001‐1; Beyotime).

### BLI

BLI assays were conducted to investigate protein interactions. His‐tagged PlpE (50 μg/ml) was immobilized on an anti‐His biosensor (ForteBio). ABCF2 at various concentrations (18.75–600 nM) was applied, and interactions were analyzed using an Octet RED96 BLI system (ForteBio).

### Western blot analysis

Before Western blot analysis, cells underwent various treatments: (1) Monolayers of A549 cells were inoculated with *P. multocida* HuN001, Δ*plpE*, or C*plpE* at an MOI = 200 for 12 h to examine the expression of ABCF2; (2) Monolayers of NPTr cells were inoculated with *P. multocida* HuN001 (MOI = 200) for 1, 2, 6, and 12 h to examine p‐p65; (3) NPTr monolayers were treated using an IκB/IKK inhibitor BAY11‐7082 (5 μM, Beyotime) for 24 h, followed by being inoculated with *P. multocida* HuN001, Δ*plpE*, or C*plpE* (MOI = 200) for an additional 12 h to examine p‐p65 and the expression of ABCF2; (4) The monolayers of wild‐type cells, ABCF2‐KO cells, and ABCF2‐overexpressed cells were inoculated with *P. multocida* HuN001, Δ*plpE*, or C*plpE* for 12 h to examine the level of p53, Bcl‐2, and Bax, respectively.

Subsequently, cells were lysed using cell lysis buffer for Western blot and IP with protease inhibitors, centrifuged at 12,000 rpm for 10 min, and subjected to SDS‐PAGE. Proteins were transferred to PVDF membranes (Bio‐Rad), blocked in PBST (containing 5% BSA) for 3 h at room temperature, and probed with specific antibodies overnight at 4°C to examine the expression of ABCF2 (ABCF2 polyclonal antibody [1:200; MyBioSource]), p53 (p53 monoclonal antibody [1:10,000; Proteintech]), p‐p53 (phospho‐p53 (Ser15) monoclonal antibody [1:2000; Proteintech]), Bcl‐2 (Bcl‐2 polyclonal antibody [1:2000; Proteintech]), Bax (Bax monoclonal antibody [1:10000; Proteintech]), and/or GAPDH (GAPDH polyclonal antibody [1:5000; Proteintech]). After washing using PBST, the proteins were incubated with HRP‐conjugated goat anti‐mouse IgG (H + L) (1:5000; Proteintech) or HRP‐conjugated goat anti‐rabbit IgG(H + L) (1:5000; Proteintech) for 1 h. Blots were finally visualized with ECL and quantified using ImageJ (v1.8.0). Immunoreactivity was normalized to loading controls for analysis.

### Flow cytometry

Flow cytometry assays were conducted to investigate the role of ABCF2 in p53‐dependent apoptotic signaling post *P. multocida* infection. Wild‐type cells, ABCF2‐KO cells, and ABCF2‐overexpressing cells were seeded into wells (1 × 10^5^ cells per well) of a 6‐well plate (Corning) and cultured for 12 h. Wild‐type cells and/or ABCF2‐overexpressing cells were treated with or without the p53 inhibitor pifithrin‐α (40 μM; Beyotime). Cells were then inoculated with *P. multocida* HuN001 (MOI = 200) for 12 h. Apoptotic cells were stained using an Annexin V‐FITC Apoptosis Detection Kit (Beyotime) and analyzed using a CytoFLEX Flow Cytometer (Beckman).

### qRT‐PCR

Primers used for qRT‐PCR are listed in Table S1. Total RNAs were extracted from bacterial strains at mid‐log phase. cDNAs were then synthesized using a PrimeScript RT reagent Kit with gDNA Eraser (TaKaRa), which were used as the templates to examine the transcript level of *plpE* by qPCR with primers and probes listed in Table S1. Bacterial 16sRNA was used as a reference gene.

### Mouse experiments

To evaluate PlpE's impact on the virulence of *P. multocida* to mice, 4 – 6‐week‐old SPF female mice (Kunming, China) were randomly divided into three groups, and each group contained 15 mice. Mice in different groups were given an intraperitoneal injection of *P. multocida* wild‐type strains (100 CFU per mouse), Δ*plpE* (100 CFU per mouse), C*plpE* (100 CFU per mouse), and/or PBS (100 µl per mouse), respectively. After challenging, the health conditions of mice were recorded every 2 h, and at 12 h post challenge, three mice from different groups were euthanized to collect the lungs for bacterial counting. Murine lungs were also used for histological examinations. Scoring reflecting the severity of lung damage was given following previously reported criteria^50^.

### Statistical analysis

The multiple‐*t*‐test strategy in GraphPad Prism 8.0 (GraphPad Software) was used to conduct statistical analysis. Data represent mean ± standard deviation (SD). The significance level was set at *p < 0.05, **p < 0.01, and ***p < 0.001.

## AUTHOR CONTRIBUTIONS

**Yuyao Shang:** Conceptualization; data curation; formal analysis; investigation; methodology; writing—original draft. **Fei Wang:** Conceptualization; data curation; methodology; writing—review and editing. **Menghan Chen, Zihao Wang, Hanyuan Liu, Lin Hua:** Data curation; investigation; methodology; writing—review and editing. **Huanchun Chen, Bin Wu:** Conceptualization; funding acquisition; project administration; supervision; writing—review and editing. **Zhong Peng:** Conceptualization; funding acquisition; project administration; supervision; writing—original draft; writing—review and editing.

## ETHICS STATEMENT

The mouse experiment was approved by the Ethics Committee of Huazhong Agricultural University (approval ID number: HZAUMO‐2024‐0192).

## CONFLICT OF INTERESTS

The authors declare no conflict of interests.

## Supporting information


**Table S1.** Primers, probes and oligonucleotides used in this study.

**Figure S1.** Laboratory tests verifying the deletion of *plpE*, the localization of ABCF2, and the impact of ABCF2 knockout on cell proliferation. **(A)** PCR verification for the deletion of *plpE* from *P. multocida* HuN001. **(B)** A column chart showing the examination of transcriptional level of *plpE* in *P. multocida* wild type strain (WT), the *plpE*‐deleted strain (ΔPlpE), and *plpE*‐complementary strains (CPlpE). **(C)** Immunofluorescence assay (IFA) demonstrating the expression of ABCF2 (shown in red fluorescence) in the cytoplasm of A549 cells. E‐cadherin protein (shown in green fluorescence), which is expressed on the surface of cells, was included as a control. **(D)** The growing conditions of ABCF2 knockout cells (ABCF2‐KO) and A549 cells harboring the non‐target control sgRNA were determined using the Enhanced Cell Counting Kit‐8 reagent. **(E)** Molecular docking between ABCF2 and PlpE using the structures predicted by AlphaFold3. Predicted amino acid residues crucial for interacting with ABCF2 in *P. multocida* adhesive proteins are shown in the boxes.

**Figure S2.** Quantification of Western blot bands in Figure 5D using ImageJ software. In all bar graphs, data are presented as the mean ± SD. Statistical comparisons were performed using two‐way ANOVA test. *p < 0.05; **p < 0.01; and ***p < 0.001.

## Data Availability

The mass spectrometry proteomics data have been deposited in the ProteomeXchange Consortium via the iProX partner repository under the data set identifier. Other data are available in the main text and Supporting Information.
