# Supplementary material for: Pasteurella lipoprotein E interacts with host ABCF2 to facilitate adhesion and cell apoptosis
Source: mLife. 2026 Jul 19;5(4):447–58. doi: 10.1002/mlf2.70056 (PMC13529201; doi:10.1002/mlf2.70056)
Supplement: Supplementary file 1 [file MLF2-5-447-s001.pdf]

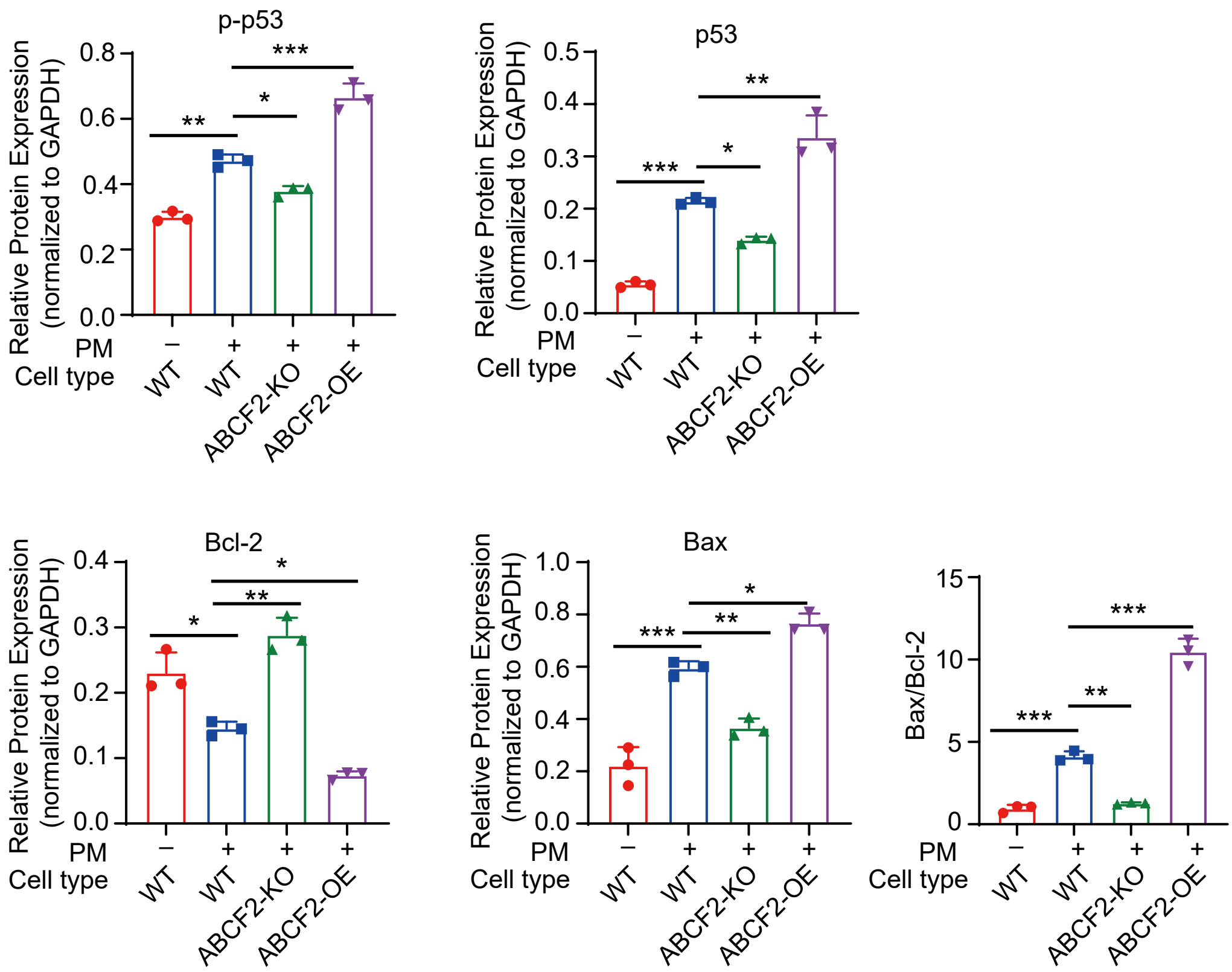

p-p53
Relative Protein Expression
(normalized to GAPDH)
0.0
0.2
0.4
0.6
0.8
**
*
***
p53
0.0
0.1
0.2
0.3
0.4
0.5
***
*
**
Bcl-2
0.0
0.1
0.2
0.3
0.4
*
**
*
Bax
0.0
0.2
0.4
0.6
0.8
1.0
***
**
*
Bax/Bcl-2
0
5
10
15
***
**
***
PM
− + + +
Cell type
WT
WT
ABCF2-KO
ABCF2-OE
