# Supplementary material for: Pasteurella lipoprotein E interacts with host ABCF2 to facilitate adhesion and cell apoptosis
Source: mLife. 2026 Jul 19;5(4):447–58. doi: 10.1002/mlf2.70056 (PMC13529201; doi:10.1002/mlf2.70056)
Supplement: Supplementary file 2 [file MLF2-5-447-s003.pdf]

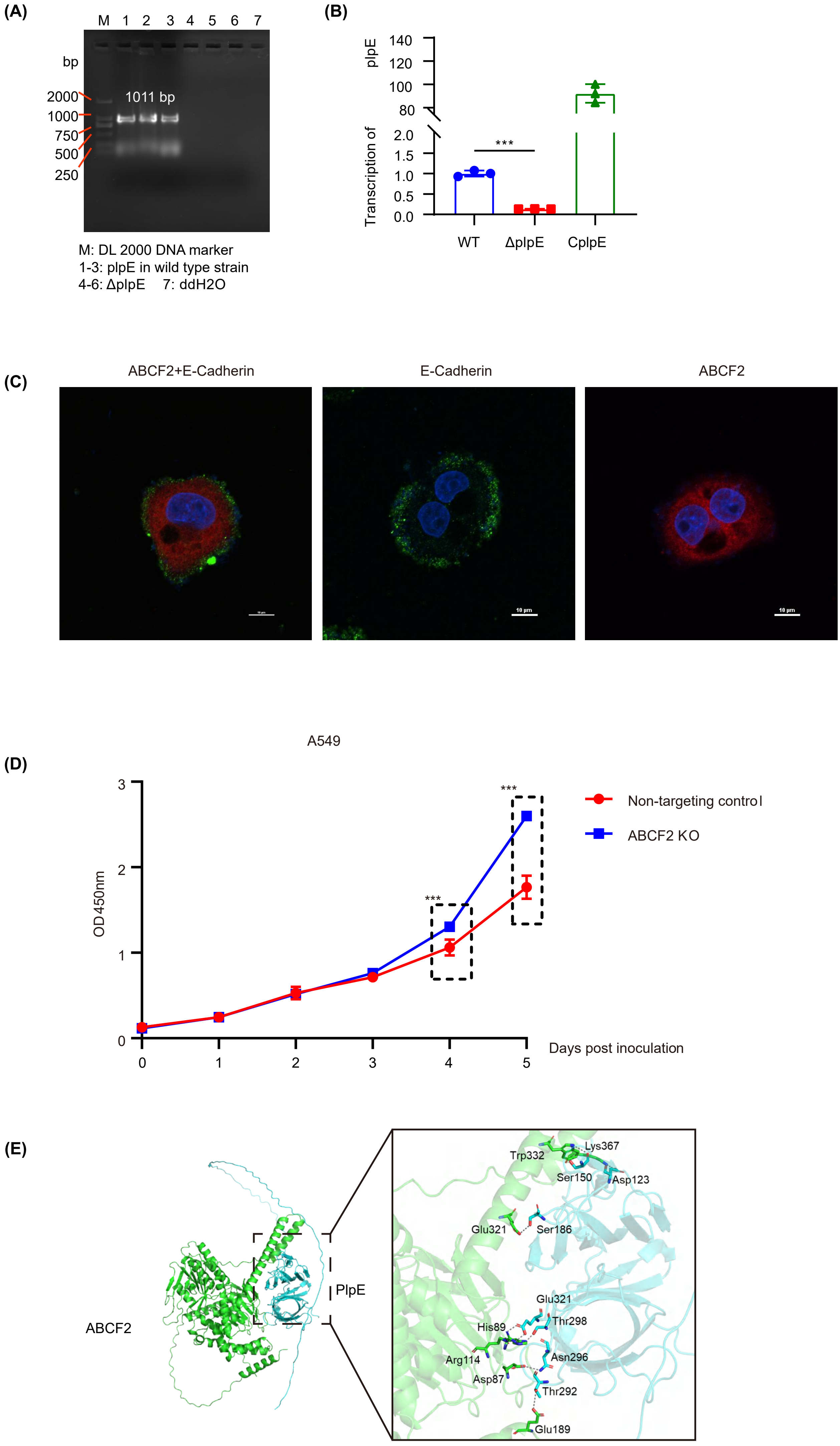
(A)
M 1 2 3 4 5 6 7
bp
2000
1000
750
500
250
1011 bp
M: DL 2000 DNA marker
1-3: plpE in wild type strain
4-6: ΔplpE 7: ddH2O
(B)
Transcription of plpE
140
120
100
80
2.0
1.5
1.0
0.5
0.0
***
WT
ΔplpE
CplpE
(C)
ABCF2+E-Cadherin
E-Cadherin
ABCF2
10 μm
10 μm
(D)
A549
OD450nm
3
2
1
0
***
***
0
1
2
3
4
5
Days post inoculation
Non-targeting control
ABCF2 KO
(E)
ABCF2
PlpE
Trp332
Lys367
Ser150
Asp123
Glu321
Ser186
Glu321
Thr298
His89
Arg114
Asn296
Asp87
Thr292
Glu189
