# Supplementary material for: Pasteurella lipoprotein E interacts with host ABCF2 to facilitate adhesion and cell apoptosis
Source: mLife. 2026 Jul 19;5(4):447–58. doi: 10.1002/mlf2.70056 (PMC13529201; doi:10.1002/mlf2.70056)
Supplement: Supplementary file 3 [file MLF2-5-447-s002.docx]

**Table S1. Primers, probes and oligonucleotides used in this study**

| **Primer** | **Sequence (5’-3’)** | **Characteristics** |
| --- | --- | --- |
| P1-F | CAGCAAATGGGTCGCGGATCCTGTAGCGGTGGTGGCGGTA | Sequence of *plpE* (cloning onto pET-28a) |
| P1-R | GTGGTGGTGGTGGTGCTCGAGTTATTGTGCTTGGTGACTTTTTTCAGCA |  |
| P2-F | TATGACCATGATTACGCCAAGCTTTGTAGCGGTGGTGGCGGTA | Sequence of *plpE* (cloning onto pPBA1101) |
| P2-R | GCCAGTGAATTCGAGCTCGGTACC TTATTGTGCTTGGTGACTTTTTTCAGCA |  |
| P3-F | CAGCAAATGGGTCGCGGATCCTGTAGCGGTGGTGGCG | Sequence of *plpE* (connecting with TurboID) |
| P3-R | GCCGCTTCCACCGCCACCTTGTGCTTGGTGACTTTTTTCAGCA |  |
| P4-F | GGTGGCGGTGGAAGCGGCGGTGGCGGAAGCATGAAAGACAATACTGTGCCTCTG | Sequence of TurboID |
| P4-R | CTCGAGTGCGGCCGCAAGCTTTCACTTTTCGGCAGACCGC |  |
| PlpE-uL | TTCCTGCAGCCCGGGGGATCCCAGGTAAGTTTTCTGAACGCAGTTGG | Upstream fragment of *plpE* |
| PlpE-uR | ATCTAGGCAGTACAAGGATAAATTACAGGACCCGACAACATGACA |  |
| PlpE-dL | ATTTATCCTTGTACTGCCTAGATCAAAAATAGGT | Downstream fragment of *plpE* |
| PlpE-dR | CGCGGTGGCGGCCGCTCTAGATCTCCTTGCTTTCCCTAAAATTTGCAAA |  |
| PlpE-oL | GCAAATAATTTACTCCGCCATAAACA | Identification of *plpE* deletion |
| PlpE-oR | GTGACCTATTTTTGATCTAGGCAGTACAAG |  |
| PlpE-iL | GGGGAAAGATCATAAGGGTTATTTTTATGT | Identification of *plpE* deletion (1101bp) |
| PlpE-iR | ATGAAAAAAATCGTTTTAAAAACAAGCTTATTGA |  |
| PlpE-L | GTCGACTGTAGCGGTGGTGGCG | Sequence of *plpE* |
| PlpE-R | CTCGAGTTATTGTGCTTGGTGACTTTTTTCAGCAAA |  |
| PlpE-F-qPCR | TTGATTGGAGTGGAAGAAGGC | qPCR for *plpE* |
| PlpE-R-qPCR | TTAGTGGCATGTAGCGGTGG |  |
| pmu-16S-F | TTCGGTCGGGAACTCAAAGG | qPCR for 16SrRNA of *P. multocida* |
| pmu-16S-R | CTCTCGCTGCCCTCTGTATG |  |
| EGFP-uL | CGGATCCATGGTGAGCAAGGGCGA | Sequence of *egfp* |
| EGFP-uR | AGCTGCCGCCACCGCTGCCGCCACCGCTGCCGCCACCCTTGTACAGCTCGTCCATGCC |  |
| PlpE-EGFP-uL | CAAGGGTGGCGGCAGCGGTGGCGGCAGCGGTGGCGGCAGCTGTAGCGGTGGTGGCG | Sequence of *plpE* for connecting *egfp* |
| PlpE-EGFP-uR | CAAGCTTTTATTGTGCTTGGTGACTTTTTTCAGC |  |
| C-PlpE-F | ACGGCCAGTGAATTCGAGCTCATGAAAAAAATCGTTTTAAAAACAAGCTTATTGATG | Sequence of *plpE* for generating complementary strains |
| C-PlpE-R | CAGGTCGACTCTAGAGGATCCTTATTGTGCTTGGTGACTTTTTTCAGCA |  |
